# Correlations between isokinetic knee torques and single-leg hop distances in three directions in patients after ACL reconstruction

**DOI:** 10.1186/s13102-021-00265-5

**Published:** 2021-04-09

**Authors:** Junya Aizawa, Kenji Hirohata, Shunsuke Ohji, Takehiro Ohmi, Sho Mitomo, Hideyuki Koga, Kazuyoshi Yagishita

**Affiliations:** 1 Department of Physical Therapy, Faculty of Health Science, Juntendo University, 3-2-12 Hongo, Bunkyo-ku, Tokyo, 113-0033 Japan; 2 Department of Rehabilitation Medicine, Graduate School of Medical and Dental Sciences, Tokyo Medical and Dental University, Tokyo, Japan; 3 Clinical Center for Sports Medicine and Sports Dentistry, Tokyo Medical and Dental University, Tokyo, Japan; 4 Department of Joint Surgery and Sports Medicine, Graduate School of Medical and Dental Sciences, Tokyo Medical and Dental University, Tokyo, Japan

**Keywords:** Hamstring, Quadriceps, Isokinetic strength, Jump-landing, Side direction, Anterior cruciate ligament

## Abstract

**Background:**

When planning rehabilitation and conditioning for performance enhancement and a return to sports after anterior cruciate ligament reconstruction, identifying the elements of physical function associated with single-leg hop is important. The purpose of this study was to clarify the relationship between single-leg hop distances in three directions and knee extensor and flexor strengths at 6 months after reconstruction.

**Methods:**

Participants were 47 patients taking part in training sessions for sports involving cutting, pivoting, and jump-landing 6 months after reconstruction using a hamstring tendon. Single-leg hop distances in 3 directions (anterior, lateral, and medial) and isokinetic concentric strengths of knee extension and flexion were assessed at an angular velocity of 60°/s and 180°/s. Simple regression analyses using Spearman’s rank correlation coefficient were performed to assess relationships between single-leg hop distances and knee strengths.

**Results:**

In the involved limb, correlations between single-leg hop distances in 3 directions and knee strengths were significant (*P* < 0.01) and correlation coefficients ranged from 0.48 to 0.65. Correlation coefficients between all single-leg hop parameters and knee extension/flexion strengths at an angular velocity of 180°/s were greater than those of 60°/s.

**Conclusions:**

In this cross-sectional study of patients who participated in sports training sessions that required jump-landings and cutting approximately 6 months after reconstruction using hamstring grafts, isokinetic knee flexor, and extensor torques were moderately to strongly associated with single-leg hop distances in lateral, medial, and anterior directions. Given these relationships, assessments and exercises for knee strength and single-leg hop distances should be planned.

## Background

Many athletes who tear the anterior cruciate ligament (ACL) undergo reconstruction and long-term postoperative rehabilitation and conditioning to improve knee function and performance, to return to sports [1]. However, after reconstruction, around 17% of elite athletes are unable to return to their original, pre-injury sports [1]. This represents a major issue for professionals involved in rehabilitation medicine and conditioning.

After reconstruction, decreases and asymmetry in single-leg hop (SLH) distance are likely to persist for a long time [2–10]. In post-reconstruction patients, decreased SLH performance is associated with lower improvements in subjective symptoms [11], and hinders a return to pre-injury sports [12–14]. These issues should be considered, especially for athletes aiming to return to sports that require jump-landing and cutting. Identification of those elements of physical function associated with SLH is important when planning strength and conditioning training for performance enhancement and a return to sports after reconstruction.

Previous studies have shown a moderate correlation between anterior SLH distance and knee extensor strength on the operated limb [5, 15–17]. To our knowledge, only the study by Sueyoshi et al. examined the relationship between anterior SLH distance and knee flexor strength in addition to knee extensor strength [17]. On the operated side in patients 6.6 months after reconstruction, they showed that the correlation coefficients between anterior SLH distance and peak torque at 180°/s of knee flexion and extension were 0.58 and 0.42, respectively [17]. In their study, 19 of 29 patients (66%) underwent reconstruction with patellar tendon. Patterns of restoration of knee extensor and flexor strength differ between patients after reconstruction using patellar tendon and after reconstruction using hamstring graft [18, 19]. Whether the results of the study by Sueyoshi et al. [17], which suggest that knee flexor strength is more related to SLH distance than knee extensor strength, are directly applicable to patients after reconstruction using hamstring graft is thus unclear.

In sports that require frequent jump-landing and cutting, not only the anterior SLH, but also lateral and medial SLH abilities are required. The relationship between anterior SLH distance and knee strength has been reported in several studies [5, 15–17]. However, the relationship between lateral and medial SLH distances and knee strength has not been analyzed and is unknown.

The purpose of this study was to clarify the relationship between SLH distances for three directions and knee extensor/flexor strength for patients participating in a sport training session 6 months after reconstruction using hamstring graft. The 6-month time point was chosen because many orthopedic surgeons decide about clearance for returning to sports that involve jump-landing and cutting at this time [20]. We hypothesized that knee extensor and flexor strength would correlate with lateral and medial SLH distances in addition to anterior SLH.

## Methods

### Participants

**Fig. 1:**
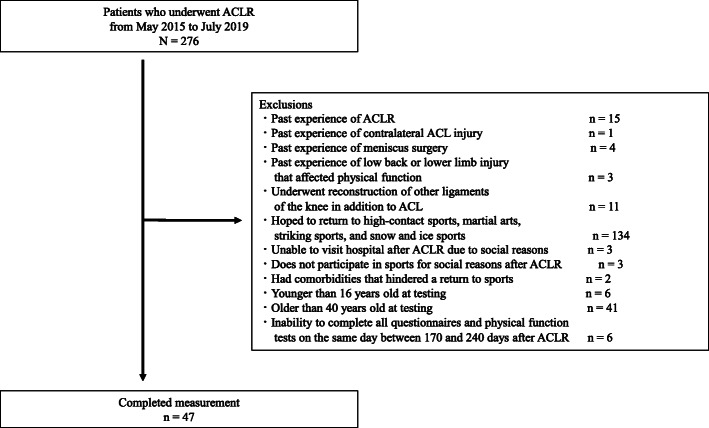
Flowchart of patients. ACL, anterior cruciate ligament; ACLR, anterior cruciate ligament reconstruction

From May 2015 to the end of July 2019, patients in this cross-sectional study were selected from the list of patients who underwent ACL reconstruction in the department of joint surgery and sports medicine at a single center (Fig. 1). Inclusion criteria were primary/unilateral anatomical double-bundle reconstruction using either hamstring tendon autograft alone or gracilis tendon harvested in addition to hamstring tendon; age ≥ 16 years and ≤ 40 years at testing; postoperative rehabilitation with the same protocol used in the sports physical therapy department; participation in sports involving cutting, pivoting, and jump-landings (basketball, soccer, volleyball, badminton, tennis, or frisbee) before injury; and participating in the training sessions of the same sport played before surgery at approximately 6 months after reconstruction [21].

Patients were excluded if they had past experience with ACL or meniscus injury or surgery on the other side; experienced injury such as muscle strains, sprains, and tendinopathy that affected physical function in the lower back or lower limb after ACL reconstruction and in the 6 months before reconstruction; undergone reconstruction of other ligaments of the knee in addition to ACL; hoped to return to high-contact sports with tackling, such as football and rugby, martial arts sports that require pairing and throwing, such as judo and wrestling, striking sports such as boxing, and snow and ice sports such as skiing and ice hockey; did not go to the hospital or participate in sports for social reasons such as relocating or becoming pregnant after reconstruction; had comorbidities that hindered a return to sports; inability to complete all questionnaires and physical function tests on the same day between 170 and 240 days after ACL reconstruction [21]. For participants under 18 years old, consent was obtained from parents and legal guardians in addition to from the participants themselves. The institutional review board at our institution approved the study, according to the Declaration of Helsinki (approval number: M2019-019). All participants provided written, informed consent.

### Postoperative rehabilitation

The postoperative rehabilitation protocol was the same for all patients [22, 23]. However, patients who underwent repair of the middle posterior segment of the meniscus were prohibited from deep squatting until 3 months after surgery [24–27]. Thirty-two patients underwent repair of the middle posterior segment of the meniscus. Patients were permitted to begin isometric quadriceps exercises as tolerated from the day after ACL reconstruction. Using a knee brace (Straighten Position Knee-Joint Immobilizer; ALCARE, Tokyo, Japan) and crutches, partial weight-bearing (20 kg) was permitted on the first day after reconstruction, and gradually increased to the maximum body mass of each patient. Use of the knee brace and crutches was discontinued at 4 weeks after reconstruction. Range-of-motion exercises from full extension to 120° of flexion were started on the second day after reconstruction. Closed kinetic chain exercises such as weight shifting and squatting were started 1–2 weeks after reconstruction. Patients were instructed to refrain from repeated knee extension training with maximum resistance near the ankle in a sitting position within the range of 10–30° of knee flexion for 3 months after surgery [28, 29]. A perturbation training program was started 3 months after reconstruction.

Running exercises were started in athletes who had cleared the criterion of limb symmetry index (LSI) of 65% of the knee extensor strength, as measured by the Biodex Multi-Joint Testing and Rehabilitation System (BDX-4; Biodex Medical Systems, New York, NY, USA) at 3 months after reconstruction. Speed and distance of running were gradually increased for joint effusion and symptoms of each patient. Once 80% of subjective full-speed running ability was achieved, athletic exercises related to the desired sporting activities were initiated with detailed instructions. All athletic exercises were specific to each patient, depending on the type of sport and position played. Participation in sports exercises with limited contact was allowed from 6 months after reconstruction, as long as the patient showed no problematic symptoms in the joint and displayed sufficient knee isokinetic flexion/extension strength at 60°/s (LSI, > 80%) and showed SLH ability (LSI, > 80%) after the specified athletic training without contact had been completed [22, 23]. Criteria for determining when to participate in the actual sport were 8 months after reconstruction [21], 90% LSI of flexion/extension strength at 60°/s [30], 90% LSI of SLH [30], 60 points on the anterior cruciate ligament-return to sport after injury scale [31, 32], and 90% of subjective running ability [21].

### Measurements

Demographic characteristics, modified Tegner activity scale score before injury, days from injury to reconstruction, days after reconstruction, and meniscus surgery procedure were taken from medical records. SLH distances and knee strength in the operated (involved) limb and non-operated (uninvolved) limb were measured on the same day, which varied by patient. The rest interval between single-leg hop tests and knee strength tests was 10 min. All physical function tests were conducted by four physiotherapists, each with more than 10 years of clinical experience in rehabilitation and conditioning of patients after ACL reconstruction.

Sex was determined based on medical records. Height and body mass were measured on the same testing day, and body mass index (BMI) was calculated. Oral questioning was used to confirm whether the knee that had undergone reconstruction was on the dominant side. The dominant limb was defined as the leg used to kick a ball to maximal distance [33]. The level of sports activity before injury was graded using the modified Tegner activity scale [34].

The date of injury and date of reconstruction were confirmed by the patient and from medical records, and the number of days from date of injury to date of reconstruction was calculated. The number of days after reconstruction was the number of days from surgery to testing.

Meniscus injuries and treatments were confirmed from detailed records of arthroscopic findings during reconstruction. The injured segment (anterior, middle, or posterior), injury type (longitudinal, radial, or horizontal) and treatment method (suture, centralization, or partial meniscectomy) were confirmed. Participants were defined as being treated regardless of the method used.

SLH distances in the three directions (anterior, lateral, and medial) were measured in random order according to previous research [21, 35]. Participants stood on one leg and were instructed to hop as far as possible and land on the same leg. The longest distance of three trials was recorded for each leg and each direction. The test was considered successful if the landing was stable. If the patient landed with early touchdown of the contralateral limb, which represented loss of balance, or took additional hops after landing, the hop was repeated. Patients were initially given a verbal description of the test and were allowed to perform as many practice trials as desired, until they felt confident about the test. Participants were allowed to use their upper limbs as desired during single-leg hops. Three trials were performed for each leg, always starting with the non-surgical limb. The rest interval between anterior, lateral and medial SLH tests was 3 min. For anterior SLH, the distance between the front end of the toe at starting position and the trailing edge of the heel at landing position was measured [36]. For lateral SLH, the distance between the lateral side of the foot at starting position and the medial side of the foot at landing position was measured. For medial SLH, the distance between the medial side of the foot at starting position and the lateral side of the foot at landing position was measured. Results are represented as the distance-to-height ratio. Total SLH distance was calculated by standardizing the total of the three-direction SLH distances by height. Intraclass correlation coefficient (ICC) case 1 was calculated to examine the reproducibility of SLH distances in three directions in the involved limb and uninvolved limb of 10 athletes who met the same inclusion criteria as in this study. To determine ICCs, SLH distance was measured 3 times in a single day and ICCs of 1–3 measured values were calculated in each direction. As a result, the ICCs of the single measurement values of the involved limb and uninvolved limb were in the range of 0.91–0.99 and 0.91–0.96, respectively, showing almost perfect reproducibility [37].

The Biodex Multi-Joint Testing and Rehabilitation System (BDX-4; Biodex Medical Systems, New York, NY, USA) was used to evaluate the isokinetic strength of the knee in extension/flexion. To minimize compensatory movements during testing, participants were seated and secured with padded straps around the thigh, pelvis, and torso. The femoral condyle of the tested limb was aligned with the rotation axis of the torque meter. Participants performed 3–5 repetitions of submaximal knee extension/flexion to familiarize themselves with the testing motion. To determine the strength of knee extension/flexion, participants performed 5 consecutive concentric contractions of extension/flexion at 60°/s and 180°/s. Peak torque within the five trials was extracted and normalized by body mass. Prior to strength measurements, participants were verbally instructed to repeat the cycle of extending and bending the knee as strongly and quickly as possible over the entire range of motion until the end of measurement was declared. No verbal commands were provided during measurements. The uninvolved limb was tested first. Five minutes of rest was provided between familiarization and strength tests. The rest interval between strength tests at 60°/s and 180°/s was 5 min. The knee strength test at 60°/s was performed first, followed by the test at 180°/s. Results were represented by peak torque-to-weight ratio. The test-retest reliability of concentric peak torque for the knee using the Biodex System was high to very high [38–42].

### Statistical analysis

Sample size was calculated from the correlation coefficient between anterior SLH distance and knee extension strength on the operated side in previous studies of patients after ACL reconstruction [15]. A priori power analysis revealed that at least 42 participants would be required to achieve an effect size of 0.475, with an alpha value of 0.05 and a power of 0.90 [43]. Due to potential attrition, a total of 47 subjects were recruited and tested in the current investigation. The normality of each variable was confirmed by the Shapiro-Wilk test. Simple regression analyses using Spearman’s rank correlation coefficient were performed to clarify the relationship between SLH distance and knee strength. The correlations were classified as weak (correlation coefficient [*r*] < 0.30), medium (≥ 0.30 but < 0.50), and strong ≥ 0.50) [43]. All data were analyzed with the Statistical Package for the Social Sciences for Windows (version 21.0; IBM Corp., New York, NY, USA). Values of *P* < .05 were used to indicate statistical significance.

## Results

Table 1 shows the participant characteristics. Median age was 20 years and 68% were female. Median modified Tegner activity scale score before injury was 7.0. Mean time from reconstruction to testing was 185.0 days.

**Table 1:** Participant data

Sex (male/female)	15/32
Age at testing (years)	20.0 (7.0) [19.9–23.4]
Height (cm)	165.0 ± 0.1 [162.6–167.3]
Body mass (kg)	60.1 ± 9.1 [57.4–62.7]
Body mass index (kg/m^2^)	22.0 ± 2.1 [21.4–22.6]
Involved limb (left/right)	28/19
Dominance of involved limb (dominant/non-dominant)	23/24
Modified Tegner activity scale score before injury	7.0 (2.0) [7.5–8.1]
Days from injury to reconstruction	55.0 (60.0) [64.2–122.1]
Autograft (hamstring tendon/gracilis + hamstring tendon)	44/3
Meniscus treated/non-treated	32/15
Days from reconstruction to testing	185.0 ± 16.4 [180.1–189.7]
Sports involved in (basketball/soccer/volleyball/badminton/tennis/frisbee)	9/16/12/8/1/1

Values are mean ± standard deviation or median (interquartile range) [95% confidence interval]. All other values are presented as number of patients or limbs

Table 2 shows values of SLH distance and knee strength. SLH distance-to-height ratio in the involved limb for the three directions ranged from 46.7 to 64.5%. Total SLH distance of the involved limb and uninvolved limb were 165.6% and 188.1%, respectively.

**Table 2:** Single-leg hop distance and knee strength

	Involved limb	Uninvolved limb
Anterior SLH (% height)	64.5 (22.8) [61.7–70.6]	76.0 (25.7) [71.5–79.8]
Lateral SLH (% height)	46.7 (19.0) [45.2–52.4]	57.0 (16.8) [53.8–61.1]
Medial SLH (% height)	53.3 (19.6) [49.2–56.6]	57.3 (18.1) [56.0–63.4]
Total SLH (% height)	165.6 (63.0) [156.8–178.9]	188.1 (52.2) [182.0–203.7]
KES 60°/s (Nm/kg)	2.0 (0.6) [2.0–2.2]	2.4 (0.8) [2.3–2.6]
KES 180°/s (Nm/kg)	1.4 (0.4) [1.4–1.6]	1.7 (0.4) [1.6–1.9]
KFS 60°/s (Nm/kg)	1.1 (0.3) [1.0–1.1]	1.2 (0.3) [1.1–1.4]
KFS 180°/s (Nm/kg)	0.8 (0.3) [0.7–0.9]	0.9 (0.3) [0.9–1.0]

Values are median (interquartile range) [95% confidence interval]. *SLH* Single-leg hop distance, *Total* Sum of hop distances in three directions, *KES* Knee extension strength, *KFS* Knee flexion strength

Table 3 shows bivariate correlation coefficients between variables in the involved limb and uninvolved limb. In the involved limb, correlations between SLH distance and knee extension strength were significant, with correlation coefficients in the range of 0.51–0.65. These correlations were considered strong. Correlations between SLH distance and knee flexion strength were also significant, with correlation coefficients in the range of 0.48–0.59. These correlations ranged from medium to strong. In the uninvolved limb, correlations between SLH distance and knee extension strength were significant, with correlation coefficients in the range of 0.51–0.64. These correlations were strong. Correlations between SLH distance and knee flexion strength were significant, with correlation coefficients in the range of 0.51–0.62; these correlations were also strong. In the involved limb, correlation coefficients between all SLH parameters and knee extension/flexion strength at an angular velocity of 180°/s were greater than those at 60°/s.

**Table 3:** Spearman’s rank correlation coefficients between single-leg hop distance and knee strength

Involved limb	SLH			
	Anterior	Lateral	Medial	Total
KES 60°/s	0.51** [0.26–0.70]	0.52** [0.27–0.70]	0.55** [0.31–0.72]	0.54** [0.30–0.72]
KES 180°/s	0.62** [0.41–0.77]	0.65** [0.45–0.79]	0.59** [0.37–0.75]	0.65** [0.45–0.79]
KFS 60°/s	0.54** [0.30–0.72]	0.53** [0.29–0.71]	0.48** [0.22–0.67]	0.55** [0.31–0.72]
KFS 180°/s	0.57** [0.34–0.74]	0.58** [0.35–0.74]	0.54** [0.30–0.72]	0.59** [0.37–0.75]
Uninvolved limb	SLH			
	Anterior	Lateral	Medial	Total
KES 60°/s	0.61** [0.39–0.76]	0.58** [0.35–0.74]	0.64** [0.43–0.78]	0.64** [0.43–0.78]
KES 180°/s	0.53** [0.29–0.71]	0.51** [0.26–0.70]	0.59** [0.37–0.75]	0.57** [0.34–0.74]
KFS 60°/s	0.53** [0.29–0.71]	0.56** [0.33–0.73]	0.54** [0.30–0.72]	0.57** [0.34–0.74]
KFS 180°/s	0.51** [0.26–0.70]	0.62** [0.41–0.77]	0.60** [0.38–0.76]	0.61** [0.39–0.76]

Values are Spearman’s rank correlation coefficients [95% confidence interval]. *SLH* Single-leg hop distance, *Total* Sum of hop distances in three directions, *KES* Knee extension strength, *KFS* Knee flexion strength. **P < 0.01

## Discussion

This cross-sectional study analyzed associations between SLH distances for three directions and knee strength in patients participating in sports training sessions requiring jump-landing and cutting 6 months after reconstruction using hamstring graft. Results showed that knee extension/flexion strength were moderately to strongly associated with lateral and medial SLH distances in addition to anterior SLH distance.

In a previous study of patients 6.6 months after reconstruction, the correlation coefficients of anterior SLH distance and knee extension/flexion strength at an angular velocity of 180°/s were 0.42 and 0.58, respectively, in the involved limb [17]. This result indicates that anterior SLH distance is more strongly associated with knee flexor strength than with extensor strength. In this study, correlation coefficients between anterior SLH distance and knee extensor and flexor strength at an angular velocity of 180°/s were 0.62 and 0.57, respectively, indicating both correlations were strong. Therefore, the relationship between anterior SLH distance and knee extension/flexion strength may have differed between this and the previous study [17]. In the previous study, 66% of patients had undergone reconstruction using patellar tendon graft [17]. On the other hand, subjects in this study were only patients who underwent reconstruction with a hamstring graft. Postoperative symptoms and recovery patterns of knee extension/flexion strength differ depending on the type of graft used for reconstruction [18, 19].

In a study of patients 8.3 months after reconstruction, the correlation coefficients of anterior SLH distance and knee extensor strength at angular velocities of 60°/s and 180°/s were 0.36 and 0.59, respectively, in the involved limbs [15]. Also in this study, in the involved limb, knee strength at an angular velocity of 180°/s was more strongly related to the anterior SLH distance than that at 60°/s for both extension and flexion. The results of this study supported the previous study. The strength of the correlation between knee extensor strength and anterior SLH distance at an angular velocity of 60°/s in the involved limb differed between the previous study and the present investigation (correlation coefficients: 0.36 vs. 0.51, medium vs. strong, respectively). In the previous study, 69% of patients had undergone reconstruction using a patellar tendon graft [15]. Subjects in this study were limited to patients who underwent reconstruction with a hamstring graft. Postoperative symptoms and recovery patterns of knee strength are known to differ according to the type of graft used for reconstruction [18, 19]. Such differences may have contributed to differences in correlations between anterior SLH distance and knee extension strength between this and the previous study.

In this study, the correlation coefficients between lateral SLH distance and knee extension/flexion strength at angular velocities of 60°/s and 180°/s were within the range of 0.52 to 0.65 in the involved limb. These correlations were strong. The correlation coefficients between medial SLH distance and knee extension/flexion strength at angular velocities of 60°/s and 180°/s were within the range of 0.48–0.59 in the involved limb, and these correlations were medium to strong. Similar results were obtained with uninvolved limbs. In the involved limb, knee strength at an angular velocity of 180°/s was more strongly related to lateral and medial SLH distances than those at 60°/s for both extension and flexion. No previous reports have shown a relationship between lateral and medial SLH distance and knee strength in patients undergoing reconstruction.

In the current study, the correlation coefficients between the total of anterior, lateral, and medial SLH distances and knee extension/flexion strength at angular velocities of 60°/s and 180°/s were within the range of 0.54–0.65 in the involved limb and these correlations were strong. In the involved limb, knee strength at an angular velocity of 180°/s correlated more strongly with total SLH distance than that at 60°/s for both extension and flexion. The relationship between isokinetic knee extensor strength and vertical jump height of both legs at different angular velocities has been analyzed for healthy female basketball players [44]. In the previous study, isokinetic knee extensor strength at an angular velocity of 180°/s correlated more strongly with vertical jump height than that of 90°/s [44]. In this study, in the involved limb, the correlation coefficients between all SLH parameters and knee extension/flexion strength at an angular velocity of 180°/s were greater than those of 60°/s. One possible reason for this result is that the knee extension/flexion movements and muscle contraction speeds required by SLH are relatively close to those at an angular velocity of 180°/s.

Previous studies have analyzed the relationship between anterior SLH distance and strength of both knee extension and flexion, selecting patients without limiting the type of graft used in reconstruction [17]. However, no previous studies have shown a relationship between distances for single-leg hop in three directions (anterior, medial and lateral), and knee extensor and flexor muscle strength, only for athletes who have undergone hamstring graft reconstruction. Rehabilitation and conditioning professions are required to plan instructional content after understanding the surgical information of the athlete and the characteristics of postoperative functional recovery. These results may help in planning conditioning for athletes participating in training sessions 6 months after reconstruction with hamstring grafts, aiming to return to the pre-injury sport. Specifically, to increase SLH distances in the three directions of anterior, lateral, and medial, measuring isokinetic knee extension/flexion strength at an angular velocity of 180°/s and increasing these weight ratios is important.

Several reports have analyzed the relationship between LSI of anterior SLH distance and that of knee muscle strength in post-reconstruction patients [4, 15, 45, 46]. Some of these studies concluded that the association between the two variables was either not significant or weak, and the results were inconsistent between studies. LSI is an index obtained by dividing the value of the involved limb by the value of the uninvolved limb. Since function of the non-operative side is reduced after reconstruction, if LSI is used as an index of functional recovery, knee strength and SLH ability of the reconstructive side may be overestimated [47]. The weight ratio, not the LSI of knee extension strength, is reportedly one of the factors that hinder the return to sports after reconstruction [12]. For these reasons, planning conditioning while checking the standardized value of knee muscle strength by body mass is important to improve SLH distance for returning to sports after reconstruction.

This study has some limitations. Fourteen participants were under 18 years old (16 years old, *n* = 6; 17 years old, *n* = 8). Since adolescents are physiologically different from adults [48], these age group differences may have contributed to greater heterogeneity in the results. Since subjects in this study participated in sports that require frequent single-leg jump, landing and cutting in multiple directions, there are limits to the applicability of the results to athletes who participate in sports that do not require much jump-landing and cutting. This study excluded athletes aiming at a return to collision sports, martial arts, striking sports, or snow and ice sports. Research into correlations between SLH distances and knee strength for those sports is currently being performed separately. Values and relationships of SLH distance and knee strength may differ between dominant and non-dominant legs, but these were not analyzed separately [49]. Patterns of recovery for postoperative knee strength differ depending on whether the graft used in reconstruction is a hamstring or patellar tendon [18, 19]. As this study targeted only patients after reconstruction with hamstring grafts, generalization of the findings to patients after reconstruction with patellar tendon graft remains limited.

## Conclusions

In this cross-sectional study of patients who participated in sports training sessions that require jump-landings and cutting approximately 6 months after reconstruction using hamstring grafts, isokinetic knee flexor, and extensor torques were moderately to strongly associated with single-leg hop distances in the lateral, medial, and anterior direction. Based on these relationships, conditioning knee strength and single-leg hop should be planned for patients after ACL reconstruction.

## Data Availability

The datasets used and/or analyzed during the current study are available from the corresponding author on reasonable request.

## Abbreviations

ACL: Anterior cruciate ligament
SLH: Single-leg hop
LSI: Limb symmetry index
ICC: Intraclass correlation coefficient
